# Scientific Contributions of Population-Based Studies to Cardiovascular Epidemiology in the GWAS Era

**DOI:** 10.3389/fcvm.2018.00057

**Published:** 2018-06-07

**Authors:** Wolfgang Lieb, Ramachandran S. Vasan

**Affiliations:** ^1^Institute of Epidemiology, Kiel University, Kiel, Germany; ^2^Framingham Heart Study (FHS), Framingham, MA, United States; ^3^Section of Preventive Medicine and Epidemiology, Boston University School of Medicine, Boston, MA, United States

**Keywords:** GWAS (genome-wide association study), population, genetic variation, genetic predisposition to disease, risk prediction

## Abstract

Longitudinal, well phenotyped, population-based cohort studies offer unique research opportunities in the context of genome-wide association studies (GWAS), including GWAS for new-onset (incident) cardiovascular disease (CVD) events, the assessment of gene x lifestyle interactions, and evaluating the incremental predictive utility of genetic information in apparently healthy individuals. Furthermore, comprehensively phenotyped community-dwelling samples have contributed to GWAS of numerous traits that reflect normal organ function (e.g., cardiac structure and systolic and diastolic function) and for many traits along the CVD continuum (e.g., risk factors, circulating biomarkers, and subclinical disease traits). These GWAS have heretofore identified many genetic loci implicated in normal organ function and different stages of the CVD continuum. Finally, population-based cohort studies have made important contributions to Mendelian Randomization analyses, a statistical approach that uses genetic information to assess observed associations between cardiovascular traits and clinical CVD outcomes for potential causality.

## What Are Key Features of Population-Based Cohort Studies?

As a brief introduction, we would like to highlight important design features of population-based studies. As opposed to hospital-based referral samples, population-based epidemiological studies examine community-dwelling or random samples from the general population. As such, study participants are not selected based on a given disease, but rather to represent the general population of the areas sampled, so that observations from such a sample are generalizable to the underlying source population. It has to be kept in mind, though, that the response rate of some landmark cohort studies is rather low [e.g., 5,5% for the UK Biobank (1)], which increases the potential for selection bias (2). Furthermore, most population-based studies are longitudinal studies that are re-examining their participants every few years so that repeated measures of several traits are available and trajectories over time (and their genetic underpinning) can be assessed, as opposed to analyses of single occasion measurements of select traits in typical referral samples. Thus, population-based cohort studies include many individuals free of the disease of interest at the beginning of the study, but who might develop the condition of interest over the course of the study. Therefore, population-based cohort studies are ideal to study risk factors and intermediate traits for the development of chronic disease conditions and to estimate measures of disease incidence (3, 4).

Third, many population-based cohort studies perform [Bibr B5]deep physiological/clinical and molecular phenotyping of their study participants (5). For example, comprehensive physiological, biochemical, subclinical, and clinical measurements are obtained on the participants using highly standardized methods. Similarly, clinical endpoints are adjudicated in a comprehensive and highly standardized process, which enhances the accuracy and validity of endpoint data from population-based cohort studies. The molecular characterization may include the assessment of common and rare genetic variation and other OMICs measurements, such as epigenomics, transcriptomics, lipidomics, proteomics, and metabolomics (5). These key features of population-based studies allow specific research questions to be addressed in the context of genome-wide association studies (GWAS). For example, the detailed phenotyping allows comprehensive adjustments and mediation analyses in order to delineate whether an observed association between a genetic variant and cardiovascular outcomes is independent of traditional risk factors and whether traditional risk factors or biomarkers might mediate the observed association. Overall, population-based studies have made a substantial contribution to scientific discoveries in the GWAS era. A few illustrative highlights of such findings from cohort studies are described below.

## Reference Sample for Genetic-Epidemiological Analyses

Since many community-dwelling samples are representative of the general population, population-based studies have served as reference (“control”) samples for many genetic case-control analyses. In essence, genetic case-control studies compare allelic frequencies of genetic variants in prevalent cases (patients who have the disease of interest when they are sampled) and controls. Ideally, the control sample captures the distribution of the exposure (in this case, the allele frequencies of putative genetic variants) in the source population from which the cases were derived (6). Therefore, population-based studies have provided controls for genetic case-control studies of a broad spectrum of traits, including myocardial infarction (MI)/coronary artery disease (CAD) (7), stroke (8, 9), and dilated cardiomyopathy (10). Importantly, as detailed below, GWAS might reveal different results depending on whether prevalent or incident cases are being analyzed.

## GWAS Analyses for a Broad Spectrum of Phenotypic Traits and Biomarkers Along the Cardiovascular Disease Continuum

The broad and highly standardized phenotyping of their study participants has allowed many different contributions of population-based cohort studies to GWAS. Specifically, researchers from population-based studies have performed and contributed to numerous GWAS for traits along the cardiovascular disease continuum, including traditional CVD risk factors [e.g., lipids (11), blood pressure (12, 13), and glycemic traits (14)], circulating cardiovascular biomarkers [e.g., B-type natriuretic peptide (BNP) (15), C-reactive protein (16), troponin (17), aldosterone, renin concentration, renin activity (18), adipokines (19), and fibrinogen levels (20)], and subclinical cardiovascular disease traits [such as indices of left ventricular structure and function (21, 22), carotid intima media thickness [IMT] (23), and coronary artery calcification (24)]. [Bibr B25][Bibr B21][Bibr B26][Bibr B15]Of note, cardiac function can be assessed by different modalities, including e.g., ECG, echocardiography, MRI/CT and circulating biomarkers; and genome-wide genetic analyses have been conducted for various of these traits, including ECG parameters (25), echocardiographic traits (21, 22) and MRI measures of cardiac structure and function (26), as well as relevant biomarkers (15). 

[Bibr B27]It is important to keep in mind that community-based samples (as opposed to clinical samples with established disease) include many individuals free of CVD at the time of inclusion in the study so that population-based cohort studies offer great opportunities to study the development of cardiovascular disease conditions over the adult life course (27), including very early (clinically asymptomatic) stages of the disease process and the genetic underpinning of these early stages. Thus, the above-mentioned GWAS have described to what extent different stages along the CVD continuum are associated with genetic variation and which genes might be involved.

Furthermore, given the large proportion of apparently healthy individuals in population-based cohort studies (as opposed to clinical samples), these studies conducted GWAS of many traits that reflect relatively normal organ function, including biomarkers of cardiac structure and systolic and diastolic function (21, 22). These studies provided important insights how physiological organ function is influenced by genetic variation, and how organ dysfunction might contribute to different disease processes (21, 22).

## Assessment of Gene X Lifestyle Interactions

It is an important and growing area of research to quantify the contribution of genes and of different lifestyle factors (and their interactions) to inter-individual variation in cardiovascular risk factor levels and disease risk. Since well phenotyped cohort studies usually have comprehensive genetic data and detailed lifestyle information available, population-based studies represent an ideal setting to study gene x lifestyle interactions. The interaction of a genetic risk score (based on 50 SNPs) and a lifestyle score (including information on smoking, obesity, physical activity, and diet) on the incidence of CAD has been analyzed in several large community-based cohorts (28). Key observations from these analyses were that (i) both scores, the genetic risk score and the lifestyle score, were independently associated with the risk of incident CVD and that (ii) a favorable lifestyle was associated with an almost 50% reduction in the relative risk for CAD, as compared to those with an unfavorable lifestyle profile (28). This reduction in the relative risk of CAD by a favorable lifestyle was observed in individuals with high genetic risk, but also in individuals with low and intermediate genetic risk (28). Very similar observations were made in more than 270.000 participants of the UK Biobank, when a polygenic risk score, representing 314 BP-associated loci, as well as a slightly different lifestyle score (including information on body mass index, healthy diet, sedentary lifestyle, alcohol consumption, smoking, and urinary sodium excretion levels) were related to different BP traits and to incident CVD (29). Both, the genetic risk score as well as the lifestyle score were associated with BP traits and incident CVD. Importantly, a favorable lifestyle as compared to an unfavorable lifestyle was associated with substantially lower average BP values in all categories of genetic risk (low, intermediate, high) and with an about 30% lower relative risk for incident CVD [Bibr B29](29). 

The same lifestyle score as in Reference (28) was used in a sample of young women (aged 25 to 40 years) from the Dutch Lifelines cohort to assess the contribution of rare and common genetic variation and of lifestyle factors to very low (≤1st age- and sex-specific percentile) and very high (≥99% age- and sex-specific percentile) levels of LDL-C. The study revealed that about two thirds of the women with very low LDL-C levels had a likely genetic cause (either a relevant mutation in an established gene for monogenic hypocholesterolemia or a very low polygenic risk score), whereas the lifestyle score (28) was not statistically significantly associated with low LDL-C concentrations (30). In cases with hypercholesterolemia, however, an unfavorable lifestyle seems to be more relevant. Only about 40% of the women had a genetic cause (relevant mutations in genes for monogenic familial hypercholesterolemia) or predisposition (high polygenic risk score) for high LDL-C; and of the women without genetic cause for hypercholesterolemia, more than half of women displayed an unfavorable lifestyle profile (30).

Community-based studies have also been involved in studying uncommon loss-of-function variants that may offer insights into function of variants. For example, (gain-of-function) mutations in the PCSK9 (proprotein convertase subtilisin/kexin type 9) serine protease gene were initially identified in families with autosomal dominant hypercholesterolemia (31). Subsequently, loss-of-function mutations were reported in individuals with low circulating low-density lipoprotein (LDL) cholesterol levels (32). Analyses in population-based studies revealed that low-frequency sequence variants in the *PCSK9* gene and a *PCSK9* genetic score were associated with lower circulating LDL cholesterol levels and reduced risk of cardiovascular events in the general population (33, 34). Recently, PCSK9 inhibitors have been tested in randomized controlled trials (35).

## The Genetic Underpinning of Change in Cardiovascular Traits Over the Life Course

Due to the availability of repeated measures over time, cohort studies are also suitable to explore the genetic underpinning of changes in cardiovascular risk factors over time, and of the progression of subclinical CVD traits longitudinally. For example, a GWAS for carotid IMT measured at different time points over a 10-year period has recently been published (36). Furthermore, several researchers assessed the association of risk factor-associated genetic variants with trajectories of the respective risk factor over the life course. For example, BMI-associated genetic variants have been related to repeated measures of BMI over time (37). Interestingly, BMI in childhood and adulthood were associated with different sets of single nucleotide polymorphisms (SNPs) (37), respectively, consistent with the concept that genetic effects on risk factors might be age-dependent. In line with this concept, genetic linkage analyses for BMI provided evidence for age-dependent effects of select genetic loci (38).

On a parallel note, a genetic risk score consisting of 29 SNPs was not only associated with blood pressure and hypertension prevalence at baseline, but also with new-onset hypertension and change in blood pressure over the life course in a large Swedish cohort study (39).

## GWAS for Incident Disease Conditions

The longitudinal character of population-based cohort studies allows genetic variation to be studied in relation to disease incidence. For example, population-based cohort studies have facilitated GWAS for incident heart failure (40), incident stroke (41) and incident MI/coronary heart disease (CHD) (3). Interestingly, GWAS for incident MI/CHD (3) reported partially discrepant results as compared to GWAS using prevalent CAD cases (7). As an example, the chromosome 9p21 locus – consistently replicated in case-control GWAS for CAD/MI (7, 42) – provided only modest evidence for association in a GWAS for incident MI/CHD within the CHARGE consortium (3). Of note, the CHARGE consortium (Cohorts for Heart and Aging Research in Genomic Epidemiology) was founded to coordinate joint GWAS analyses of several traits in large population-based cohort studies and to provide opportunities for mutual replication efforts (43).

It is well known that analyses based on prevalent disease cases and those based on incident cases might reveal different results if the association between an exposure and the disease outcome differs by disease severity or disease duration (a phenomenon referred to as prevalence-incidence bias) (44). In order to be included in a case-control study as prevalent MI/CAD case, MI patients have to survive the acute event until they are sampled. Given that MI is still associated with substantial case fatality (45, 46), case-control studies are likely enriched for MI/CAD survivors with rather long survival (3). Thus, alleles associated with prevalent CAD in case-control analyses could be related to the risk of developing the CAD event, but could also be related to the chances of surviving the acute CAD event. In line with this concept, the CAD risk allele at the 9p21 locus was associated with longer survival after MI in several population-based cohorts within CHARGE (3).

## Impact of Genetic Variation on Risk Prediction

Furthermore, community-based prospective cohorts allow assessing whether genetic information improves risk prediction models beyond traditional risk factors. It was, indeed, one of the main motivations of the human genome project to use genetic information to predict disease risks in healthy individuals and to predict the response to a given therapy among patients. Several analyses conducted in various population-based cohorts assessed whether genetic variation – e.g., in an aggregated form as risk scores – improved performance measures of risk prediction models for a first CVD event, including discrimination, calibration, and reclassification (47–50). Although the results from individual studies vary, in most cases, the genetic risk scores displayed clear statistically significant associations with CVD endpoints, but improvements in discrimination (e.g., C-statistics; integrated discrimination improvement) and reclassification (e.g., net reclassification index) were more modest (47, 48) and some studies did not provide evidence for improvement in these performance metrices beyond traditional risk factors (49, 50).

## Mendelian Randomization Analyses for Cardiovascular Traits

Genetic information in population-based cohort studies has also been used to assess causality between cardiovascular risk factors or circulating biomarkers and cardiovascular outcomes (incident CVD events) using instrumental variable analyses, a statistical approach referred to as Mendelian Randomization (MR) (51–53). This term, MR, refers to the random assortment of alleles of a given locus at meiosis (51, 52). Thus, if a genetic locus (or a genetic risk score) is strongly associated with circulating biomarker levels or with risk factor levels, individuals are “randomized” to genetically determined high or low biomarker/risk factor levels (51, 52, 54). If the biomarker/risk factor is causally related to CVD, this difference in genetically determined higher or lower biomarker/risk factor levels should translate into corresponding quantitative differences in disease risk (51, 52, 54). Therefore, in addition to the association between the genetic variant and the risk factor/biomarker of interest, MR analyses also assess the associations between the risk factor/biomarker and incident CVD as well as between the genetic variant and incident CVD (52); the two latter analyses are facilitated by population-based cohort studies. By using genetic information as instrumental variable for the biomarker/risk factor of interest, MR analyses try to avoid two important limitations of observational studies, reverse causality and confounding (54, 55). Using MR analyses in population-based samples, several traits along the CVD continuum and biomarkers have been tested for potentially causal relations with incident CVD, including high-density lipoprotein (HDL) cholesterol (53), C-reactive protein (56), lipoprotein(a) (57), and many others. It has to be kept in mind, though, that instrumental variable analyses can be affected by different types of selection bias. For example, such analyses might be biased, if a genetic variant is related to mortality, and MR analyses are conducted in an elderly sample (58, 59).

## Conclusion

Population-based studies have substantially improved our understanding of the genetic architecture of normal and abnormal organ function, CVD risk factors, circulating biomarkers, subclinical disease, and overt CVD traits over the life course. Furthermore, they were essential in exploring gene x lifestyle interactions and in evaluating genetic variation in the context of risk prediction models for incident CVD. In addition, population-based cohort studies provided great opportunities to conduct GWAS for incident CVD events, such as MI, stroke and heart failure, and thereby, to overcome classic limitations of case-control GWAS including prevalence-incidence bias. Finally, population-based cohort studies used genetic information as instrumental variables to assess whether cardiovascular risk factors or biomarkers are causally related to clinical CVD (Mendelian Randomization analyses).

## Author Contributions

WL and RV wrote the article together.

## Conflict of Interest Statement

The authors declare that the research was conducted in the absence of any commercial or financial relationships that could be construed as a potential conflict of interest.
